# M. Civiale on Structure of the Urethra

**Published:** 1842-11-26

**Authors:** 


### *Academy of Sciences, Paris, October* 10, 1842.

*M. Civiale on Stricture of the Urethra.*—In this memoir the author examines the following questions:—What is the organic lesion which constitutes stricture?—what the effects of neglected stricture?—and what the inconveniences resulting from the modes of practice described in surgical works? The answers which M. Civiale gives to the above questions are:

1. It has been generally laid down, that stricture of the urethra depends on some accidental production developed on the lining membrane of the canal and diminishing its calibre, and it has been proposed to destroy this production. I have shown that instead of being seated in the lining membrane, the diseased condition exists underneath the mucous membrane, which remains healthy; hence, the usual modes of treatment adopted are faulty, inasmuch as they cannot reach the seat of the disease without destroying the lining membrane of the urethra.

2. Stricture was commonly supposed to occupy the membranous portion of the urethra much more frequently than any other part. I have shown that the disease is rarely, if ever, seated in the membranous portion, and, consequently, that caustics or cutting instruments should not be directed to this part of the canal.

3. Hitherto the same mode of treatment has been applied to stricture, without any regard being had to its seat; but I have shown that the mode of treatment should vary according as the stricture may occupy the meatus, the spongy portion, or the sub-pubic curve—the three most frequent seats of the disease.

4. The attention of surgeons has been chiefly directed to the alterations which take place at the strictured point itself; but I have shown that these are of secondary importance, and that the changes which take place *behind* the seat of the stricture are infinitely more deserving the attention of the practitioner. These are chronic inflammation of the urethra, neck and cavity of the bladder, ulceration, abcess, infiltration of urine, dilatation of the membranous and prostatic portions of the urethra, and of the prostatic and seminal ducts; in a word, a long catalogue of affections implicating the prostate and neck of the bladder, and requiring the utmost attention of the surgeon, though too often neglected for the original disease which occasioned them.

